# 从中国胸外科医师3年年会征稿看我国胸外科的学术发展

**DOI:** 10.3779/j.issn.1009-3419.2016.06.01

**Published:** 2016-06-20

**Authors:** 逊 张

**Affiliations:** 300000 天津，天津市胸科医院 Tianjin Chest Hospital, Tianjin 300000, China

中国医师协会胸外科医师分会自2007年成立以来，已历经两届分会委员会。协会除力争在众多方面为广大胸外科医师做好服务外，另一重要内容就是主办一年一度的胸外科医师年会，本届分会自委员会2014年成立以来，就年会学术作了较大改革，以图吸引更多同行参与交流。下面就3年来年会的征稿、发言论文的出版做一汇报，供大家参考，并呼吁大家做好来年年会投稿的准备。

## 1 加强继续教育

继续教育是医师分会年会的主要宗旨，2014年后更加得到加强，以2014年年会为例：①大会设热点问题知名专家讲座；②在保留传统“肺外科”和“食管外科”分会场的同时，另设“胸腺外科”专场，并由胸腺研究小组编译了国外同行的共识性文章发表于2014年的《中国肺癌杂志》；③对各分会场入选发言的原创性研究，特邀该领域知名专家进行专题点评，点评内容主要涉及与该研究相关的历史现状、未来前景及对作者研究内容的评价与建议等，从而达到多方位继续教育的目的。

本届年会除秉承上述理念外，还约请了国内知名专家就《早期肺癌》与《食管癌热点问题》撰写继续教育文章，分别发表于2016年的《中国肺癌杂志》及《临床外科杂志》，供大家参考。

## 2 重视原创研究

原创性研究历来是学科创新的灵魂，自2014年以来协会通过发布通知、电话催稿、特邀布置等方式，致力于挖掘全国各有关单位出彩的研究。因此，从2013年原创投稿不足百篇的状态跃升到今年的200余篇，在重视原创、涵盖重点学科及顾及全国覆盖的原则上，经过多轮审稿，精挑细选出优质稿件作为各会场的主题发言，并在此基础上约请本专题资深专家进行专业点评，以达到继续教育、带动作者、提高原创性研究水平的目的。

## 3 争取论文发表

自2014年会起，分会即组织专家对分会场发言稿件、壁报稿件及所有投稿稿件进行追踪发表，本着作者自愿、数据真实、稿件质量过关的原则，协会在帮助作者论文发表方面做了大量工作，包括对基层医师一对一逐一修改，将几乎全部有意愿发表的稿件在国际及国内知名刊物刊发。在此，要特别感谢《中国肺癌杂志》、《中华胃肠外科杂志》、《*Journal of Thoracic Disease*》（*JTD*）等杂志的大力协助，尤其感谢《中国肺癌杂志》、《临床外科杂志》等著名杂志将今年年会的教育性文献在会议期间同期发表。今年年会稿件出现了各大型杂志供不应求的现象。

